# Temporal Trends in Carbapenem-Resistant *Acinetobacter baumannii* Isolation Rates at a Regional Hospital in Central Taiwan, 2020–2024

**DOI:** 10.3390/antibiotics15050486

**Published:** 2026-05-11

**Authors:** Ying-Ju Chen, Tze-Kiong Er

**Affiliations:** 1 Division of Laboratory Medicine, Asia University Hospital, Asia University, Taichung 41354, Taiwan; 050478@tool.caaumed.org.tw; 2 Department of Medical Laboratory Science and Biotechnology, Asia University, Taichung 41354, Taiwan; 3 Department of Nursing, Asia University, Taichung 41354, Taiwan

**Keywords:** carbapenem-resistant *Acinetobacter baumannii* (CRAB), antimicrobial resistance (AMR), epidemiological trends, surveillance study, Taiwan

## Abstract

**Background/Objectives**: Carbapenem-resistant *Acinetobacter baumannii* (CRAB) is a priority nosocomial pathogen with limited therapeutic options and high attributable mortality, particularly in intensive care units (ICUs). Whether the pandemic-era rise in institutional CRAB burden resolved, persisted, or stabilized at a sustained high level remains incompletely characterized at the single-hospital level. **Methods:** We conducted a retrospective observational study of all non-duplicate clinical *A. baumannii* complex isolates recovered at a 450-bed regional teaching hospital in central Taiwan between January 2020 and December 2024. Isolates were classified as CRAB based on non-susceptibility (intermediate or resistant) to imipenem or meropenem. Annual and monthly isolation rates, ICU versus non-ICU distribution, and temporal trends were analyzed. **Results:** Among 582 *A. baumannii* complex isolates, 340 (58.4%) were classified as CRAB. The annual CRAB isolation rate differed significantly across the study period (*p* = 0.0099), rising sharply from 44.0% (51/116) in 2020 to 63.9% (76/119) in 2021 and subsequently remaining between 58.3% (60/103) and 63.2% (67/106) through 2024. Monthly isolation rates varied widely (16.7–100.0%) without a consistent seasonal pattern. The proportion of CRAB isolates originating from ICU settings varied year to year, rising from 45.1% (23/51) in 2020 to a peak of 67.4% (58/86) in 2022 and then declining to 49.3% (33/67) in 2024, without a statistically significant overall trend (*p* = 0.054). **Conclusions:** These findings suggest that CRAB isolation rates remained at a sustained elevated level after the acute pandemic phase and highlight ICUs as an important focus for institution-level infection prevention and antimicrobial stewardship efforts.

## 1. Introduction

Carbapenem-resistant *Acinetobacter baumannii* (CRAB) has emerged as one of the most consequential nosocomial pathogens of the twenty-first century, frequently associated with elevated morbidity, prolonged hospitalization, and high attributable mortality [1]. The World Health Organization has designated carbapenem-resistant *A. baumannii* as a critical priority pathogen for research and development of new antimicrobial agents, reflecting both the severity of infections it causes and the progressive narrowing of effective therapeutic options [2]. The clinical burden is concentrated disproportionately in intensive care units (ICUs), where patients requiring mechanical ventilation, central venous catheterization, and prolonged broad-spectrum antimicrobial exposure provide ideal conditions for CRAB acquisition and transmission [3]. The spectrum of infection is broad, encompassing ventilator-associated pneumonia, bloodstream infection, urinary tract infection, and complicated skin and soft tissue infection, and the emergence of multidrug-resistant and extensively drug-resistant strains has further complicated empirical therapy [4,5,6].

In Taiwan, the incidence of *A. baumannii* complex infections has markedly increased since the early 1990s, now representing a leading cause of hospital-onset bacteremia [7]. The proportion of isolates resistant to carbapenems rose dramatically from 3.4% in 2002 to 58.7% in 2010 [8]. In a prior investigation, carbapenem resistance was detected in 78.8% of *A. baumannii* strains, highlighting the substantial resistance burden in clinical isolates [9]. According to the Taiwan Healthcare-associated Infection and Antimicrobial Resistance Surveillance Report (2024 Q4) [10], the prevalence of CRAB in ICU-associated infections increased between 2015 and 2024 in both medical centers (from 62.7% to 71.5%) and regional hospitals (from 77.0% to 79.8%). In 2024, the highest CRAB rates were reported in the Eastern (87.0%) and Central (85.9%) regions. The rising burden of CRAB represents a significant challenge to patient safety and hospital infection control efforts within Taiwan’s healthcare system [11]. Further supporting these findings, a large-scale study conducted through the Antimicrobial Testing Leadership and Surveillance (ATLAS) program analyzed 2674 *A. baumannii* isolates collected from 13 countries in the Asia–Pacific region between 2012 and 2019, revealing an overall carbapenem resistance rate of 71.7%, with prevalence ranging from as low as 2.8% in Japan to as high as 88% in South Korea [12].

The COVID-19 pandemic introduced additional complexity to the already concerning trajectory of antimicrobial resistance. Increased ICU occupancy, staff redeployment, changes in personal protective equipment practices, and higher empirical antimicrobial use during the acute pandemic phase have been associated with increased CRAB transmission in multiple healthcare settings. In our previous work, we reported a multi-pathogen antimicrobial resistance snapshot at the same institution, comparing a single pre-pandemic year (2019) with a 25-month pandemic period (September 2020–September 2022) across six clinically important organisms [13]. That analysis demonstrated an increase in CRAB prevalence during the pandemic period but, by design, could not determine whether this represented a transient fluctuation or the emergence of a sustained endemic pattern. In addition, the prior study did not assess detailed temporal trends or the distribution of CRAB between ICU and non-ICU settings.

The present study extends this work through continuous five-year surveillance of CRAB among clinical *A. baumannii* complex isolates at a regional teaching hospital in central Taiwan (January 2020–December 2024). The objectives were to (i) describe annual trends in CRAB isolation rate across different pandemic phases; (ii) evaluate month-to-month variability and assess the presence of seasonal patterns; and (iii) examine the relative contribution of ICU and non-ICU settings to the overall CRAB burden. By providing longer-term, institution-level data with temporal and ward-specific resolution, this study aims to offer additional insight into CRAB dynamics in a real-world hospital setting and to support ongoing infection prevention and antimicrobial stewardship efforts.

## 2. Results

### 2.1. Overall CRAB Burden and Annual Trend

The study included 582 *A. baumannii* complex isolates collected between 2020 and 2024, of which 340 (58.4%) were classified as CRAB. Detailed annual distributions of CRAB and non-CRAB isolates, together with the total AB complex denominator for each year, are provided in Table 1; the annual totals of AB complex isolates remained relatively stable across the study period (116, 119, 138, 103, and 106 isolates in 2020 through 2024, respectively), providing a relatively stable denominator for year-over-year comparison of CRAB isolation rates. Stratification of the 340 CRAB isolates by specimen source identified sputum as the predominant specimen type, accounting for 195 isolates (57.4%, 195/340), followed by urine (52, 15.3%, 52/340), pus (45, 13.2%, 45/340), and blood (34, 10.0%, 34/340). Catheter tips (8, 2.4%, 8/340) and body fluids (6, 1.8%, 6/340) contributed the remaining isolates.

**Figure 1:**
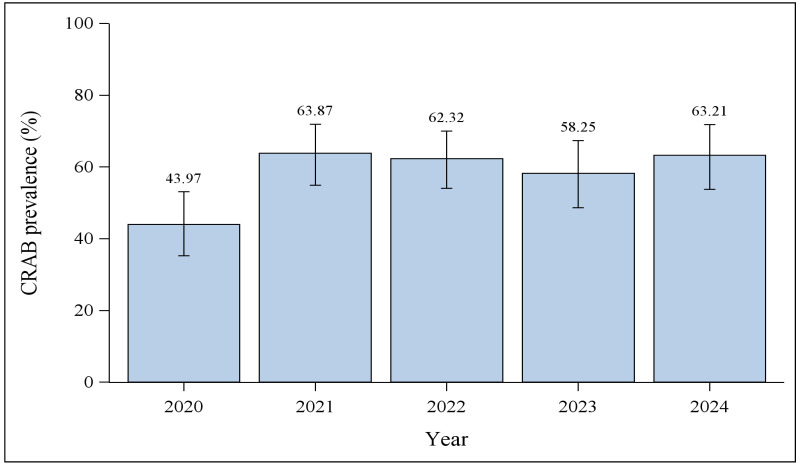
Annual isolation rate of CRAB among *A. baumannii* complex isolates (2020–2024). The bar chart illustrates the annual proportion of CRAB isolates out of the total *A. baumannii* complex identified each year. Error bars represent 95% confidence intervals for the annual CRAB isolation rates, calculated using the number of CRAB isolates as the numerator and the total number of *A. baumannii* complex isolates as the denominator. A statistically significant difference in CRAB isolation rate was observed across the five years (*p* = 0.0099). The isolation rate increased sharply between 2020 (44.0%, 51/116) and 2021 (63.9%, 76/119). Following this sharp rise, the rate remained at a sustained elevated level, ranging between 58.3% (60/103) and 63.2% (67/106) in the final three years (2022–2024).

Analysis of annual isolation rates showed a significant association between year of isolation and CRAB isolation rate (*p* = 0.0099). As shown in Figure 1, the CRAB isolation rate increased markedly from 44.0% (51/116) in 2020 to 63.9% (76/119) in 2021. This sharp rise was followed by a persistently high isolation rate over the subsequent three years. Between 2022 and 2024, CRAB rates remained relatively stable, ranging from 58.3% (60/103) in 2023 to 63.2% (67/106) in 2024, indicating a sustained elevated baseline.

It is important to place the 2020 value in the context of pre-pandemic data from the same institution. Our group’s previously published multi-pathogen survey [13] reported a CRAB prevalence of 46% among 148 AB complex isolates recovered in 2019, the most recent pre-pandemic year available. When compared with the 2019 pre-pandemic rate reported in our previous study (46%), the 2020 rate of 44.0% was similar, whereas the 2021 rate of 63.9% represented a clear increase above the pre-pandemic and early-pandemic baselines.

### 2.2. Monthly Variation and Seasonal Assessment

**Figure 2:**
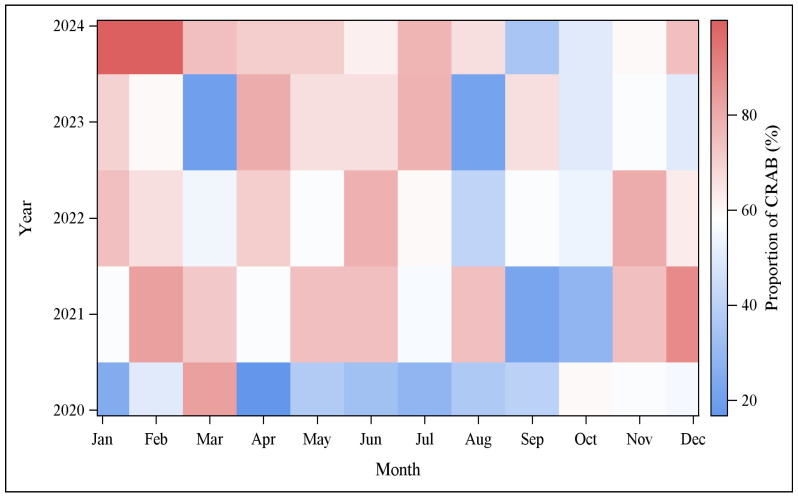
Heatmap of monthly CRAB isolation rate, 2020–2024. The heatmap displays monthly CRAB isolation rate across the five-year surveillance period at Asia University Hospital, with calendar months on the *x*-axis and study years on the *y*-axis. Color intensity represents the proportion of CRAB isolates among all *A. baumannii* complex isolates identified in each month, with darker shading indicating higher isolation rate. The monthly numbers of CRAB isolates and total AB complex isolates corresponding to each cell are provided in Supplementary Table S1, together with the calculated monthly CRAB isolation rate. The visualization highlights substantial month-to-month variability without evidence of a consistent seasonal pattern. Cells based on fewer than ten total AB complex isolates should be interpreted with caution due to small-denominator instability.

Monthly CRAB isolation rates varied considerably over the five-year study period (Figure 2; monthly counts of CRAB and total AB complex isolates for all 60 months are provided in Supplementary Table S1). Isolation rates ranged from a low of 16.7% in April 2020 to 100.0% in January and February 2024. In the latter months, all recovered *A. baumannii* isolates were carbapenem-resistant; however, these values should be interpreted with caution, as they were based on a small number of isolates (January: 6/6; February: 2/2). Marked month-to-month fluctuations were particularly evident during periods of higher overall isolation rates, most notably between 2022 and 2024. For instance, in 2023, monthly CRAB isolation rates ranged from 20% in March to 80% in April. Despite this pronounced variability, no consistent seasonal trend was observed, such as recurring increases during specific summer or winter months.

The absence of seasonal variation should be interpreted with caution and is consistent with the hospital-acquired nature of CRAB transmission in settings where the principal reservoirs are climate-controlled clinical environments. Because the majority of CRAB isolates in this study originated from ICU and other inpatient settings maintained under year-round temperature and humidity control, an absence of seasonal signal is the expected pattern rather than an independent finding, and should not be over-interpreted as evidence regarding transmission mechanism.

### 2.3. Distribution by Location of Isolation and Trend Analysis

Analysis of the 340 CRAB isolates demonstrated that the largest proportion of isolates was recovered from inpatient specimens. Overall, 307 isolates (90.3%, 307/340) were recovered from hospitalized patients, encompassing both ICU and non-ICU wards. Within this inpatient population, specimens submitted from intensive care units accounted for the largest single share, contributing 189 isolates (55.6%, 189/340) over the five-year study period.

Annual analysis by specimen submission location (Table 2) showed year-to-year variation in the proportion of CRAB isolates originating from the ICU. The ICU contribution was lowest in 2020 (45.1%, 23/51) and increased in 2021 (51.3%, 39/76), followed by a pronounced rise in 2022, when specimens submitted from ICU settings accounted for approximately two-thirds of all CRAB isolates (67.4%, 58/86). Although the ICU remained the predominant source of isolates in 2023 (60.0%, 36/60), this proportion decreased in 2024 (49.3%, 33/67).

The overall pattern across the five years describes an increase in the ICU contribution during the middle years of the study (2022–2023), followed by a decline in 2024 toward a level similar to that observed in 2020. The overall difference in ICU contribution across the study years did not reach statistical significance (*p* = 0.054). As noted in the Methods, these classifications reflect the location at which specimens were submitted and do not establish acquisition site; non-ICU isolates may include patients with prior ICU exposure during the same or earlier hospitalization, and the observed distribution should be interpreted as a descriptor of the surveillance signal rather than as proof of ward-level acquisition.

## 3. Discussion

The present study provides five-year institutional surveillance of CRAB at a regional teaching hospital in central Taiwan, extending our group’s earlier multi-pathogen report [13] and adding continuous temporal resolution, ward-level stratification, and three additional years of data through the post-acute-pandemic period. Three main observations emerge. First, CRAB isolation rates rose from a pre-pandemic and early-2020 level of approximately 44–46% to a sustained elevated baseline of 58–64% between 2021 and 2024. Second, the 2020 value was marginally lower than the 2019 pre-pandemic figure reported in our prior study [13], indicating that the 2020-to-2021 increase should be interpreted in relation to both the pre-pandemic baseline and the early-pandemic 2020 value. Third, specimens submitted from intensive care units contributed the majority of CRAB isolates over the full study period (55.6%, 189/340), though the ICU contribution varied year to year without a statistically significant trend.

These observations are consistent with reports describing increased CRAB burden during the COVID-19 pandemic. Multiple studies have reported increases in CRAB transmission in hospital settings during the acute pandemic period, attributed to a combination of elevated ICU occupancy, staff redeployment, altered infection control workflows, and increased empirical antimicrobial use. A retrospective study by Itani et al. reported a 30-day mortality rate of 71.2% among patients with *A. baumannii* infections, with inappropriate initial antimicrobial therapy, mechanical ventilation, and thrombocytopenia identified as significant predictors of death, underscoring the urgent need for timely treatment and strengthened infection control measures [14]. Sustained surveillance therefore remains essential to track institutional CRAB dynamics and to inform stewardship interventions [15].

The COVID-19 pandemic may have influenced CRAB epidemiology in hospital settings. Pascale et al. reported a 5.5-fold increase in CRAB infections in intensive care units (ICUs) during the pandemic period [16]. Consistent with this observation, our previous study at the same institution reported a CRAB prevalence of 46% among 148 *A. baumannii* complex isolates in 2019 and 65% during the 25-month pandemic period from September 2020 to September 2022, representing a 19-percentage-point increase [13]. In the present five-year surveillance study, the overall CRAB isolation rate was 58.4% (340/582), with significant annual variation (*p* = 0.0099). The annual CRAB isolation rate was 44.0% (51/116) in 2020, increased to 63.9% (76/119) in 2021, and remained persistently elevated through 2024. These findings suggest that the pandemic-period increase in CRAB isolation rates did not fully return to the pre-pandemic level during the post-acute-pandemic period.

CRAB is an important healthcare-associated pathogen, particularly in intensive care units (ICUs), where it presents major challenges for both treatment and infection control. Previous studies have shown that CRAB outbreaks occur frequently in ICU settings, often among patients receiving mechanical ventilation or prior antibiotic therapy, and are associated with poor clinical outcomes [17]. Multidrug-resistant *A. baumannii* is widely recognized as a major ICU pathogen because of its association with severe infections, limited therapeutic options, prolonged hospitalization, and increased mortality [18,19]. Jiang et al. further highlighted the growing challenge of CRAB in ICU settings, including the emergence of resistance to both carbapenems and colistin [20]. In our study, the proportion of CRAB isolates originating from the ICU increased from 48.8% (62/127) in 2020–2021 to 59.6% (127/213) in 2022–2024 (Table 2). The substantial contribution of ICU-submitted specimens to the overall CRAB isolate pool supports the prioritization of intensive care units as a focus for continued surveillance and infection prevention efforts. The predominance of respiratory specimens (sputum: 57.4%, 195/340) among CRAB isolates is consistent with the recognized importance of respiratory tract specimens in CRAB surveillance among hospitalized and critically ill patients.

The absence of a consistent seasonal pattern in monthly CRAB isolation rates is compatible with the predominantly healthcare-associated nature of CRAB in hospital settings. Because most CRAB isolates in this study originated from ICU and other inpatient settings, which are maintained under relatively stable indoor environmental conditions throughout the year, the absence of a seasonal signal should be interpreted cautiously and should not be considered evidence of a specific transmission mechanism. Short-term monthly fluctuations may reflect a combination of small-number statistical variation, particularly in months with fewer than ten isolates, local cluster events, and variations in patient acuity and case mix rather than broader climatic influences.

Several limitations of this study should be acknowledged. First, this was a single-center retrospective analysis conducted at a regional teaching hospital, and the findings should be interpreted as reflecting the experience of this specific institution rather than as representative of the broader regional or national epidemiology of CRAB in Taiwan. Second, ward classification reflects the location of specimen submission rather than the location of organism acquisition; non-ICU isolates may include patients with prior ICU exposure during the same or earlier hospitalization. In addition, ward-stratified denominators for all AB complex isolates were not available in the finalized LIS dataset; therefore, location-specific CRAB isolation rates within ICU and non-ICU settings could not be calculated. Third, the analysis was based on laboratory surveillance data and did not include adjudication of isolates as representing true infection versus asymptomatic colonization or contamination; therefore, CRAB isolation rates reported here should not be equated with CRAB infection incidence. Fourth, patient-level clinical data, including specimen indication, prior antibiotic exposure, length of hospital stay, prior ICU admission, total laboratory specimen submission volume, and clinical outcomes, were not available for this analysis and are required for a complete assessment of clinical impact. Fifth, additional laboratory characterization of isolates was not performed; specifically, neither molecular profiling of resistance mechanisms and clonal lineage nor a full phenotypic antibiogram beyond carbapenems was conducted, and these represent priorities for future work. Future multicenter studies incorporating patient-level clinical data, chart-adjudicated infection classification, molecular epidemiology, and broader phenotypic characterization are needed to build a more comprehensive picture of CRAB transmission and resistance dynamics in Taiwanese regional hospitals.

Despite these limitations, the findings of this study have practical relevance for institutional infection prevention and antimicrobial stewardship. The observation that CRAB isolation rates remained at a persistently elevated level through 2024, rather than returning to pre-pandemic levels, suggests that sustained surveillance and targeted intervention remain necessary. The continued prominence of ICU-submitted specimens as the dominant source of CRAB isolates supports the prioritization of intensive care units as a focus for continued surveillance, targeted contact precautions, environmental cleaning protocols, and antimicrobial stewardship initiatives aimed at reducing inappropriate carbapenem exposure.

## 4. Materials and Methods

### 4.1. Study Design and Setting

This retrospective observational study was conducted at Asia University Hospital, a 450-bed regional teaching hospital in central Taiwan. The hospital provides comprehensive medical services, including intensive care and infectious disease units, and its microbiology laboratory processes diagnostic specimens from hospitalized inpatients (including intensive care and general wards) and from hospital outpatient clinics. All non-duplicate clinical isolates of the *Acinetobacter baumannii*/*calcoaceticus* complex (AB complex) collected between 1 January 2020, and 31 December 2024 were included.

Isolates were obtained from routinely collected clinical specimens, including blood, urine, respiratory tract samples, and wound swabs, and processed in the hospital’s microbiology laboratory. Species identification and antimicrobial susceptibility testing (AST) were performed using the Phoenix 100^®^ automated system (BD Diagnostics, Sparks, MD, USA), following standardized laboratory procedures as previously described [21]. Carbapenem non-susceptibility was defined as an intermediate (I) or resistant (R) result for imipenem or meropenem, and isolates meeting this criterion were classified as CRAB.

This study was approved by the Institutional Review Board of China Medical University Hospital (IRB No. CMUH114-REC1-198). As only anonymized laboratory data were analyzed, the requirement for informed consent was waived.

### 4.2. Data Collection

Microbiological data for this study were retrieved retrospectively from the hospital’s electronic laboratory information system (LIS). Only non-duplicate isolates were included, defined as the first AB complex isolate per patient during the study period, in order to avoid inflated counts from repeated sampling of the same patient. For each included isolate, the data extracted comprised the date of specimen collection, the specimen type, the carbapenem susceptibility result (CRAB or non-CRAB), and the clinical ward of origin, which was used to classify each isolate as originating from an ICU, a non-ICU inpatient ward, or a non-inpatient setting.

No formal institution-wide revisions to written infection prevention and control (IPC) policies, routine clinical sampling protocols, or critical care unit structure were documented during the 2020–2024 study period; however, temporal trends should be interpreted in the context of pandemic-related healthcare system pressures. Throughout the study period, the microbiology laboratory followed standard operating procedures for culture processing, organism identification, antimicrobial susceptibility testing, and result reporting.

A subset of the 2020–2022 isolates included in the present analysis was previously included in our group’s multi-pathogen antimicrobial resistance survey at the same institution [13]. The earlier study reported aggregate carbapenem-resistant *A. baumannii* rates for two time windows: a pre-pandemic reference year (2019, *n* = 148 AB complex isolates, 46% resistant) and a 25-month pandemic period (September 2020–September 2022), evaluated alongside five other clinically important organisms. The present study differs from that earlier report in scope, time frame, and analytical resolution: it is focused specifically on CRAB, applies strict per-patient deduplication, provides continuous annual and monthly trend analysis, extends surveillance three years beyond the prior window through December 2024, and incorporates ward-level stratification that was not examined in the prior report.

A total of 582 AB complex isolates met the inclusion criteria, of which 340 were classified as CRAB. Annual and monthly CRAB isolation rates were calculated as the proportion of CRAB isolates among all AB complex isolates identified in each period, expressed as a percentage. Descriptive statistics were used to summarize counts and proportions.

The present analysis was based on laboratory surveillance data and should be interpreted within several boundaries. First, the dataset did not distinguish CRAB isolates representing clinical infection from those representing asymptomatic colonization; such classification would require chart review and clinical adjudication, which were not performed in this study. Second, ward classification reflected the specimen submission location and may not correspond to the site of organism acquisition; therefore, isolates classified as non-ICU may include patients with prior ICU exposure during the same or preceding hospitalization. Third, the term “CRAB isolation rate” is used throughout this study to refer to the proportion of AB complex isolates meeting the carbapenem non-susceptibility criterion and should not be interpreted as the patient-level incidence of CRAB infection.

### 4.3. Bacterial Identification and Antimicrobial Susceptibility Testing

All AB complex isolates were processed and identified at the hospital’s microbiology laboratory. Initial isolation was performed using routine culture methods on eosin methylene blue (EMB) and blood agar (BAP), incubated at 35 °C for 18–24 h. Both species identification and subsequent antimicrobial susceptibility testing (AST) were carried out using the Phoenix 100^®^ automated system (BD Diagnostics, Sparks, MD, USA). This system employs modified conventional, fluorogenic, and chromogenic substrates for accurate analysis. Testing utilized the BD NMIC/ID-411, PMIC/ID-95, and SMIC/ID-2 panels. Prior to testing, pure colonies were prepared in ID broth and standardized to a 0.5 McFarland turbidity using a BD PhoenixSpec™ nephelometer (BD Diagnostics, Sparks, MD, USA), following manufacturer guidelines [22]. Susceptibility results were interpreted as sensitive (S), intermediate (I), or resistant (R) in accordance with the Clinical and Laboratory Standards Institute (CLSI) M100 guidelines in force at the time of testing (M100-Ed30 through M100-Ed34 during the 2020–2024 study period) [23]. Carbapenem non-susceptibility was defined as an intermediate (I) or resistant (R) result for imipenem or meropenem, and isolates meeting this criterion were classified as carbapenem-resistant *A. baumannii* (CRAB). This definition was applied consistently with the laboratory’s standard classification used in our group’s earlier multi-pathogen survey at the same institution.

### 4.4. Monthly Isolation Rate Analysis and Heatmap Visualization

The monthly CRAB isolation rate was calculated as the proportion of carbapenem-resistant isolates among all AB complex isolates identified each month between January 2020 and December 2024. These values were expressed as percentages.

All months were included in the analysis, regardless of isolate number, to reflect real-world laboratory surveillance conditions. However, months with low isolate counts (fewer than ten AB complex isolates) were interpreted with caution, as small denominators can produce extreme percentage values that do not reflect stable trends.

A heatmap was generated to visualize temporal variation in CRAB isolation rates across months and years. Color intensity was used to represent the magnitude of the isolation rate, facilitating identification of temporal clustering and variability. The heatmap format was selected in preference to a line graph because the study spans 60 calendar months across five years, and a two-dimensional grid allows simultaneous visualization of both within-year monthly variation and between-year trends, which would be difficult to discern in a single-axis line graph with 60 data points.

### 4.5. Statistical Analysis

Annual and monthly CRAB isolation rates were calculated as the proportion of carbapenem-resistant *Acinetobacter baumannii* complex isolates among all *A. baumannii* complex isolates identified within each corresponding period. Descriptive statistics, including counts and percentages, were used to summarize the data.

To evaluate temporal trends, the chi-square test of independence (two-sided) was applied to assess the association between study year (2020–2024) and overall CRAB isolation rate. In addition, the contribution of intensive care units (ICUs) to the CRAB burden was examined by comparing the distribution of CRAB isolates originating from ICU and non-ICU settings across study years using the chi-square test. A two-sided *p* value of less than 0.05 was considered statistically significant. All statistical analyses were performed using SAS software, version 9.4 (SAS Institute Inc., Cary, NC, USA). The heatmap was generated using PROC SGPLOT with the HEATMAPPARM statement in SAS software, version 9.4 (SAS Institute Inc., Cary, NC, USA).

## 5. Conclusions

This five-year institutional surveillance study describes the trajectory of CRAB isolation rates at a regional hospital in central Taiwan from 2020 through 2024. CRAB isolation rates increased from 44.0% (51/116) in 2020 to 63.9% (76/119) in 2021 and subsequently remained at 58.3–63.2% through 2024, without returning to the pre-pandemic level reported in our group’s earlier study [13]. Specimens submitted from intensive care units accounted for the majority of CRAB isolates across the full study period (55.6%, 189/340), although the ICU contribution varied year to year without a statistically significant trend (*p* = 0.054). These observations describe the experience of a single institution and should not be over-generalized, but they may contribute to the regional evidence base suggesting that the pandemic-era increase in CRAB burden may have persisted beyond the acute pandemic period. The findings support continued institutional surveillance of CRAB, with particular attention to specimens from intensive care settings, alongside antimicrobial stewardship efforts aimed at reducing unnecessary carbapenem exposure. Molecular characterization of stored isolates, linkage to patient-level clinical data, and adjudication of isolates as infection versus colonization should be prioritized in future studies to allow a more mechanistic interpretation of the trends reported here.

## Figures and Tables

**Table 1:** Annual distribution of CRAB and non-CRAB isolates among AB complex isolates, 2020–2024.

Year	Total AB Complex	CRAB (%)	Non-CRAB (%)	*p*-Value ^a^
2020	116	51 (44.0)	65 (56.0)	
2021	119	76 (63.9)	43 (36.1)	
2022	138	86 (62.3)	52 (37.7)	
2023	103	60 (58.3)	43 (41.8)	
2024	106	67 (63.2)	39 (36.8)	
**Total**	**582**	**340 (58.4)**	**242 (41.6)**	**0.0099**

Abbreviations: AB complex: *Acinetobacter calcoaceticus-baumannii* complex; CRAB: carbapenem-resistant *Acinetobacter baumannii* complex. ^a^ Chi-square test assessing whether the proportion of CRAB isolates differed across years.

**Table 2:** Annual contribution of the intensive care unit (ICU) to the total CRAB isolate burden, 2020–2024.

Year	Total CRAB Isolates	CRAB Isolates from ICU (%)	CRAB Isolates from Non-ICU ^a^ (%)	Non-Inpatient CRAB Isolates (%)	*p*-Value ^b^
2020	51	23 (45.1)	22 (43.1)	6 (11.8)	
2021	76	39 (51.3)	30 (39.5)	7 (9.2)	
2022	86	58 (67.4)	19 (22.1)	9 (10.5)	
2023	60	36 (60.0)	19 (31.7)	5 (8.3)	
2024	67	33 (49.3)	28 (41.8)	6 (9.0)	
**Total**	**340**	**189 (55.6)**	**118 (34.7)**	**33 (9.7)**	**0.054**

Abbreviations: CRAB: carbapenem-resistant *Acinetobacter baumannii* complex; ICU: intensive care unit. ^a^ Non-ICU included isolates from general inpatient wards. ^b^ Chi-square test assessing whether the proportion of CRAB isolates differed across years.

## Data Availability

The data presented in this study are available from the corresponding author upon reasonable request. The data are not publicly available due to institutional privacy and ethical restrictions related to laboratory surveillance data.

## Supplementary Materials

The following supporting information can be downloaded at: https://www.mdpi.com/article/10.3390/antibiotics15050486/s1. Table S1. Monthly distribution of CRAB isolates among AB complex isolates, January 2020 to December 2024.
