# Supplementary material for: Temporal Trends in Carbapenem-Resistant Acinetobacter baumannii Isolation Rates at a Regional Hospital in Central Taiwan, 2020–2024
Source: Antibiotics (Basel). 2026 May 11;15(5):486. doi: 10.3390/antibiotics15050486 (PMC13203601; doi:10.3390/antibiotics15050486)
Supplement: Supplementary file 1 [file antibiotics-15-00486-s001.zip › antibiotics-4287586-supplementary.pdf]

antibiotics

MDPI

Supplementary Materials:

Supplementary Table S1. Monthly distribution of CRAB isolates among AB complex isolates, January 2020 to December 2024.

Year	Month	Total AB complex	CRAB	CRAB / Total AB (%)
**2020**	**Jan**	4	1	1/4 (25.0)
	**Feb**	10	5	5/10 (50.0)
	**Mar**	6	5	5/6 (83.3)
	**Apr**	6	1	1/6 (16.7)
	**May**	8	3	3/8 (37.5)
	**Jun**	9	3	3/9 (33.3)
	**Jul**	14	4	4/14 (28.6)
	**Aug**	19	7	7/19 (36.8)
	**Sep**	5	2	2/5 (40.0)
	**Oct**	10	6	6/10 (60.0)
	**Nov**	7	4	4/7 (57.1)
	**Dec**	18	10	10/18 (55.6)
	**Total**	**116**	**51**	**51/116 (44.0)**
**2021**	**Jan**	7	4	4/7 (57.1)
	**Feb**	6	5	5/6 (83.3)
	**Mar**	11	8	8/11 (72.7)
	**Apr**	14	8	8/14 (57.1)
	**May**	12	9	9/12 (75.0)
	**Jun**	4	3	3/4 (75.0)
	**Jul**	16	9	9/16 (56.3)
	**Aug**	12	9	9/12 (75.0)
	**Sep**	9	2	2/9 (22.2)
	**Oct**	7	2	2/7 (28.6)
	**Nov**	12	9	9/12 (75.0)
	**Dec**	9	8	8/9 (88.9)
	**Total**	**119**	**76**	**76/119 (63.9)**
**2022**	**Jan**	8	6	6/8 (75.0)
	**Feb**	6	4	4/6 (66.7)
	**Mar**	11	6	6/11 (54.6)
	**Apr**	7	5	5/7 (71.4)
	**May**	7	4	4/7 (57.1)
	**Jun**	19	15	15/19 (78.9)
	**Jul**	15	9	9/15 (60.0)
	**Aug**	17	7	7/17 (41.2)
	**Sep**	14	8	8/14 (57.1)
	**Oct**	13	7	7/13 (53.9)
	**Nov**	10	8	8/10 (80.0)
	**Dec**	11	7	7/11 (63.6)

	**Total**	**138**	**86**	**86/138 (62.3)**
**2023**	**Jan**	10	7	7/10 (70.0)
	**Feb**	10	6	6/10 (60.0)
	**Mar**	5	1	1/5 (20.0)
	**Apr**	5	4	4/5 (80.0)
	**May**	6	4	4/6 (66.7)
	**Jun**	15	10	10/15 (66.7)
	**Jul**	14	11	11/14 (78.7)
	**Aug**	14	3	3/14 (21.4)
	**Sep**	9	6	6/9 (66.7)
	**Oct**	2	1	1/2 (50.0)
	**Nov**	7	4	4/7 (57.1)
	**Dec**	6	3	3/6 (50.0)
	**Total**	**103**	**60**	**60/103 (58.3)**
**2024**	**Jan**	6	6	6/6 (100.0)
	**Feb**	2	2	2/2 (100.0)
	**Mar**	4	3	3/4 (75.0)
	**Apr**	7	5	5/7 (71.4)
	**May**	14	10	10/14 (71.4)
	**Jun**	8	5	5/8 (62.5)
	**Jul**	9	7	7/9 (77.8)
	**Aug**	9	6	6/9 (66.7)
	**Sep**	17	6	6/17 (35.3)
	**Oct**	16	8	8/16 (50.0)
	**Nov**	10	6	6/10 (60.0)
	**Dec**	4	3	3/4 (75.0)
	**Total**	**106**	**67**	**67/106 (63.2)**
**Overall**	**Total**	**582**	**340**	**340/582 (58.4)**

Abbreviations: CRAB: carbapenem-resistant *Acinetobacter baumannii* complex; AB complex: *Acinetobacter calcoaceticus-baumannii* complex. Values represent the number of total AB complex isolates and CRAB isolates identified each month respectively. Percentages indicate the monthly CRAB isolation rate. Monthly cell percentages displayed in Figure 2 (heatmap) are calculated as the proportion of CRAB isolates among all AB complex isolates identified in each month; cells based on fewer than ten total AB complex isolates should be interpreted with caution due to small-denominator instability.
